# Correlation between Clinical manifestations, radiographic findings, and operative diagnosis of pulpal status in primary molars in 4‒8-year-old children: A cross-sectional study

**DOI:** 10.34172/joddd.025.42755

**Published:** 2025-09-30

**Authors:** Naser Asl Aminabadi, Farnaz Haji Abbas Oghli, Shabnam Mohammadzadeh, Zahra Jamali

**Affiliations:** ^1^Department of Pediatric Dentistry, Faculty of Dentistry, Tabriz University of Medical Sciences, Tabriz, Iran; ^2^Department of Oral Medicine, Faculty of Dentistry, Tabriz University of Medical Sciences, Tabriz, Iran

**Keywords:** Deciduous teeth, Decision making, Dental pulp diseases, Dental pulp test, Pediatric dentistry

## Abstract

**Background.:**

Accurate evaluation of the pulp condition is one of the greatest diagnostic challenges in pediatric dentistry. A comprehensive diagnosis of pulp status is achieved by integrating diagnostic data from multiple sources. Understanding the correlation and consistency among these various data points is important. This study aimed to determine the correlation between history-based, clinical, radiographic, and operative diagnoses.

**Methods.:**

In this cross-sectional study, 4‒8-year-old children attending the Department of Pediatric Dentistry for routine dental treatment between August 1, 2021 and July 7, 2022 were included. A total of 180 primary molars were clinically and radiographically evaluated, and their dental histories were recorded. The pulpal diagnosis was determined based on the dental history, clinical findings, and radiography. The operative diagnosis was determined during pulp therapy. Finally, the correlation was assessed by Spearman’s correlation test.

**Results.:**

The strongest correlation was observed between operative and radiographic diagnoses (r=0.831, *P*<0.001). Moreover, the correlations between clinical diagnosis and operative diagnosis and between clinical diagnosis and radiographic diagnosis were the weakest (r=0.556, *P*<0.001 and r=0.591, *P*<0.001, respectively). The correlation between the diagnosis based on history and operative diagnosis (r=0.676, *P*<0.001) was stronger than that with the clinical diagnosis (r=0.633, *P*<0.001) and radiographic diagnosis (r=0.656, *P*<0.001).

**Conclusion.:**

The findings of this study underscore the importance of integrating multiple diagnostic approaches for an accurate assessment of pulp condition. Radiographic diagnosis demonstrated the strongest reliability in assessing the pulp status. Additionally, moderate correlations between clinical, history-based, and radiographic diagnoses indicate the need for a combined diagnostic strategy approach to achieve higher accuracy and effective treatment planning.

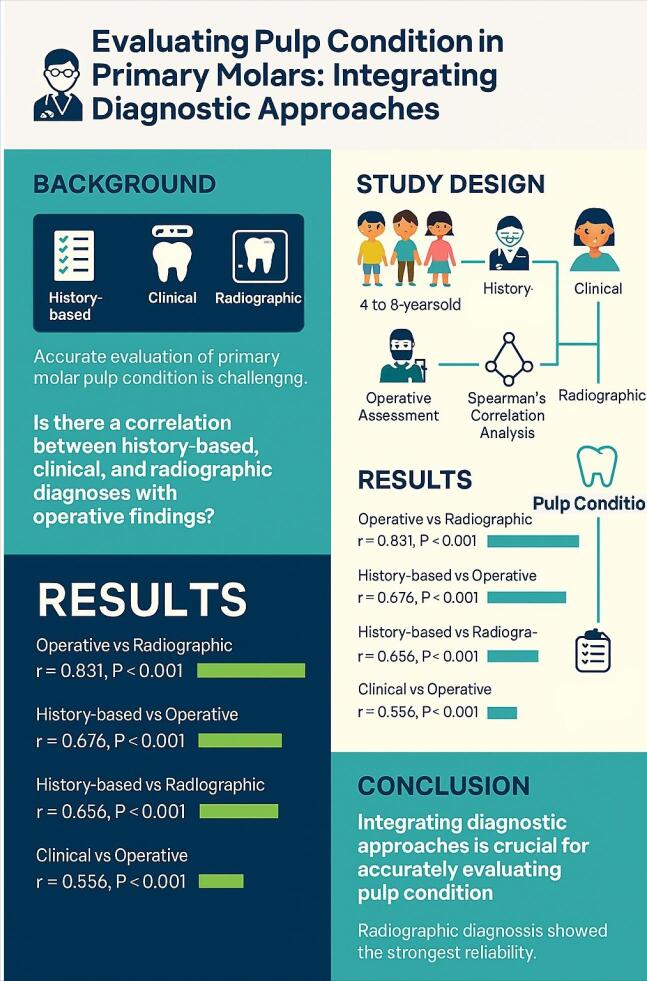

## Introduction

 Accurate pulp status diagnosis is a critical step in patient management, based on the collected data concerning its reliability and validity. The accuracy of diagnosis is based on the quality and quantity of the data collected through different methods, among which histological examination is considered the most reliable.

 Although histological examination provides the most accurate assessment of pulpal status, its application is impractical in most clinical settings. Therefore, it is essential for clinicians to thoroughly evaluate and integrate a patient’s history, clinical examination, and radiological findings to establish an accurate diagnosis of dental pulp status and determine the appropriate treatment options.^1^

 A thorough history should include assessing the characteristics of the pain and, if conceivable, an evaluation of the spontaneity of pain. However, it has been shown that a precise diagnosis of pulp status based on subjective findings, such as the existence and duration of prolonged pain in response to heat, cold, and percussion tests, cannot be achieved properly.^2^ Objective findings like acute and chronic abscess, cellulitis, excessive mobility not related to physiologic exfoliation, furcation/apical involvement in radiography, or radiographic evidence of internal/external resorption can contribute to the diagnosis of irreversible pulpitis or necrosis.^3^

 While numerous studies have confirmed the validity of different pulp sensitivity tests for permanent teeth,^4-7^ insufficient evidence is available on the validity and accuracy of pulp testing in primary teeth.^8^ The use of conventional pulp tests, which rely on patients’ understanding and cooperation, can be challenging for pediatric patients.^9^ Hori et al^8^ reported that the electric pulp test had the most sensitivity (80%) and accuracy (89%). Electric pulp testing is unreliable or relatively ineffective in primary and immature permanent teeth due to the alleged immature innervation around the odontoblasts in the pulp.^10^ Therefore, radiographic findings, excessive mobility, sensitivity to percussion, and palpation have also been used as crucial clinical aids to diagnose pulp status.

 In certain instances, definitive diagnosis can only be achieved through direct clinical assessment of the pulpal tissue. The evaluation should include the color and amount of bleeding upon direct pulp exposure; excessive bleeding or the presence of purulent exudate indicates irreversible pulpitis or pulpal necrosis.^11^ It has been demonstrated that severe pulp inflammation is associated with darker pulp bleeding color.^12^

 Therefore, making an accurate diagnosis and choosing an appropriate treatment procedure requires combining all diagnostic components to maximize the accuracy of pulp status diagnosis. However, one of the challenges in this issue is to accurately estimate the significance of data collected through different diagnostic methods. Therefore, determining the correlation between the accuracy of different diagnostic methods and the final diagnosis, as well as their validity, is of high significance. To the best of our knowledge, no study has investigated the correlation between history-based, clinical, radiographic, and operative pulpal diagnoses in primary teeth. Therefore, this study aimed to determine the extent of agreement between the perceived intensity and quality of pain, clinical assessments, radiographic findings, and operative diagnoses.

## Methods

 To calculate the sample size for the current study, the results of a study by Hori et al^8^ were considered as a reference. Considering α = 0.05 and a power of 80%, and assuming that our results would show 70% sensitivity for the electric pulp test, a minimum sample size of 137 was estimated. Assuming a dropout rate of approximately 25%, a minimum sample size of 180 was deemed necessary for adequate reliability.

 The present study was carried out in the Department of Pediatric Dentistry, Tabriz University of Medical Sciences. The participants consisted of 180 healthy children, 4‒8 years of age, who attended the Department of Pediatric Dentistry for routine dental treatment between August 1, 2021 and July 7, 2022.

 During one month, 340 children were screened during routine examinations according to the following inclusion criteria: 4‒8-year-old children with complete physical health and no confounding medical history. The participants had no history of allergies and had at least one vital primary molar tooth with deep caries, which was restorable. The children were excluded if their parents did not consent to their child being assigned to any of the approaches, suffered from any abnormal medical condition, or exhibited non-cooperative behavior during treatment appointments.

 The protocol for this cross-sectional study followed routine pulp therapy sessions, and no further procedures were imposed. All the participating children exhibited moderate oral hygiene, as defined by routine toothbrushing at least once daily. This criterion was confirmed during clinical intake to ensure consistency across the sample. At the initial visit, all children received standardized dietary instructions from the dental team. This helped reduce variability in cariogenic exposure that could influence pulpal status. Participants were recruited from a population attending regular dental check-ups at our pediatric dental clinic, which provided a relatively uniform baseline in terms of access to dental care and history of interventions. While these factors were not quantitatively analyzed, their standardization across the study helped reduce the risk of confounding bias in interpreting the correlation between clinical, radiographic, and operative findings. We have now clarified this in the revised manuscript.

 All the participants had periapical radiographs indicating a deep carious lesion requiring pulp therapy. Before starting the clinical procedure, the parents of all the participants gave informed consent and were informed about the benefits, possible risks, and potential discomfort the treatment may have.

 In the first session, the patient’s chief complaint was addressed, and all necessary periapical radiographs were obtained. The dental history was documented according to the study’s checklist. The participants

 were asked if their child had experienced any spontaneous pain, overnight pain, referred pain, or sensitivity to cold, heat, or sweet snacks in the involved tooth. The “tell-dhow-do” method was employed to familiarize the children with the dental environment, instruments, and procedures. A comprehensive clinical examination was conducted, including extraoral and intraoral assessments. The clinician assessed the following clinical signs and symptoms: sensitivity to touch or percussion, tooth mobility, presence of a sinus tract, and inflammation of the soft tissues. Pulp sensitivity tests, including thermal and electric pulp tests, were also implemented. Patients were instructed to raise their hands if they felt a cold, tingling, or uncomfortable sensation during pulp testing. A cold test was performed using a cotton pellet sprayed with dichlorodifluoromethane. The cotton pellet was sprayed until its surface became frosty and then applied to the middle third of the buccal surface of the tooth under investigation. The cotton pellet was left in contact with the tooth for either 10 seconds or until the child indicated a sensation had been felt. Any contact with the gingiva or adjacent teeth was avoided. The heat test was carried out using a hot gutta-percha stick, while the teeth were isolated with cotton. The gutta-percha stick was heated until it became soft and was applied to the middle third of the buccal surface of the tooth under investigation. A vital contralateral tooth was tested before assessing the tooth under investigation. A final-year dental student recorded the dental history and clinical findings using a checklist prepared exclusively for the study. The recorded dental history and clinical findings were then independently provided to a pediatric dentist. Each child was assigned an anonymized ID number, which was concealed from the pediatric dentist. The pediatric dentist determined the pulpal diagnosis for each patient based on an independent consideration of the dental history and clinical findings. Radiographic findings, including periapical and furcation radiolucency, periodontal ligament space widening, internal and external resorption, calcification, or pulp chamber obliteration, were documented. The pulpal diagnosis derived from the radiographic findings was made by the pediatric dentist, who was blinded to the patient’s other diagnostic data. Three possible diagnoses were assumed: reversible pulpitis, irreversible pulpitis, and necrotic pulp. The treatment plans were determined according to The American Academy of Pediatric Dentistry (AAPD) guidelines on pulp therapy for primary teeth.^3^ Based on this guideline, non-vital pulp treatment is required for teeth with irreversible pulpitis or necrosis, and teeth diagnosed with reversible pulpitis should be treated with vital pulp procedures.^13^

 In the second session, the pulp therapy procedure was performed. Following pulp exposure, the operative diagnosis was determined by a chief pediatric postgraduate dentistry student, who was blinded to the initial pulp diagnosis, under the supervision of an experienced pediatric dentist. This diagnosis was based on the quality (color) and the amount of bleeding from direct pulp tissue exposure. Extreme bleeding and darker blood color were assigned to severe inflammation, purulent exudate was indicative of pulpal necrosis, and slight oozing was suggestive of reversible pulpitis. Finally, we assessed the correlation between clinical, history-based, radiographic, and operative pulp diagnoses data.

###  Statistical analysis

 The data were analyzed using SPSS 22 (SPSS Inc., Chicago, USA). Normality of continuous variables was assessed using the Kolmogorov–Smirnov test and visual inspection of Q–Q plots. Since the data did not meet the parametric assumptions, non-parametric tests were employed.

*P *values < 0.001 were considered statistically significant, and 95% confidence intervals (CIs) were reported where applicable. The intra- and inter-examiner reliability was evaluated using the intraclass correlation coefficient (ICC), while inter-rater agreement between diagnostic methods was assessed using Cohen’s kappa statistic.

 Spearman’s rank correlation coefficient (𝑟𝑆) was used to analyze the relationship between history-based, radiographic-based, clinical-based, and operative diagnoses. Assumptions for Spearman’s test, including ordinal or continuous data and monotonic relationships, were verified before analysis. The strength of correlation was interpreted based on the absolute value of 𝑟𝑆 (Table 1).

**Table 1:** Rules for interpreting the size of Spearman’s correlation coefficient

**Size of correlation**	**Interpretation**
0.00 to 0.19 (0.00 to -0.19)	Very week(negligible) correlation
0.20 to 0.39 (-0.20 to -0.39)	Week correlation
0.40 to 0.59 (-0.40 to -0.59)	Moderate correlation
0.60 to 0.79 (-0.60 to -0.79)	Strong correlation
0.80 to 1.00 (-0.80 to -1.00)	Very strong correlation

 To minimize confounding bias, participant selection was standardized. All the children had moderate oral hygiene (brushing at least once daily), received dietary instructions at the first visit, and were recruited from a population attending regular dental check-ups. Although multivariate adjustment was not performed due to the study’s observational design and sample size, potential confounders, such as oral hygiene, diet, and visit frequency, were controlled for through the inclusion criteria and standardized clinical protocol. These limitations have been acknowledged in the discussion.

## Results

**Figure 1:**
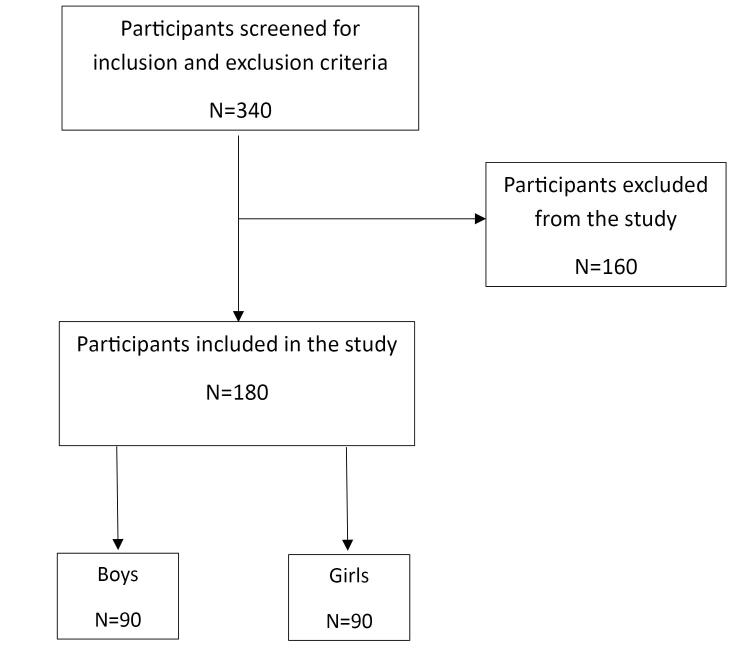
Flow diagram of number of participants

**Figure 2:**
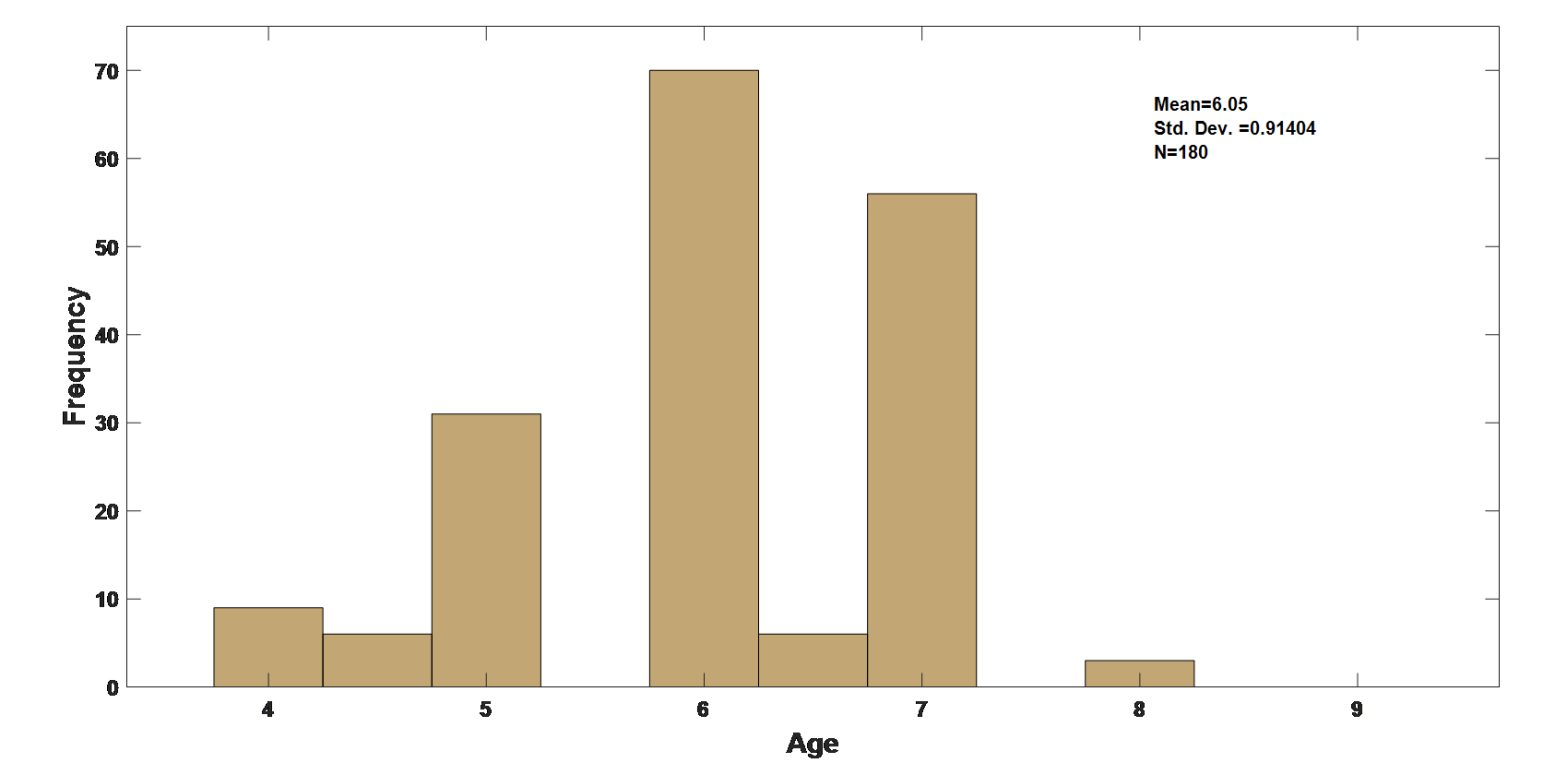
Age distribution of study participants

 A total of 180 participants were included in the study, comprising 90 girls (50.0%) (Figure 1). All the included patients were present throughout the study, and we had no missing data. The mean age of the subjects was 6.05 ± 0.91 years (Figure 2). Regarding tooth type, the study included 180 primary molars, comprising 85 maxillary molars and 95 mandibular molars. Among these, 40% were first primary molars and 60% were second primary molars. All the children were medically healthy, with no reported systemic conditions. Based on parental reports and clinical records, 70% of children had a history of dental caries, and 50% had previously received restorative treatment. None of the included teeth had undergone prior pulp therapy.

 In this study, based on the dental history provided by the parents, the pulpal diagnosis was normal in 18.3% of the cases. Reversible pulpitis and irreversible pulpitis were determined for 30.0% and 28.3% of the cases, respectively. In 23.3% of the cases, pulp necrosis was suggested based on the dental history (Table 2).

**Table 2:** Distribution of different pulpal diagnoses

	**Normal pulp**	**Reversible pulpitis**	**Irreversible Pulpitis**	**Necrosis**
Dental history	18.3%	30.0%	28.3%	23.3%
Clinical findings	13.3%	11.7%	50.0/5	25.0%
Radiographic findings	1.7%	45.0%	26.7%	26.7%
Operative diagnosis	0%	40.0%	31.7%	28.3%

 The pulpal diagnosis based on clinical findings showed normal pulp in 13.3%, reversible pulpitis in 11.7%, irreversible pulpitis in 50.0%, and necrosis in 25.0% of cases. Radiographic findings were indicative of normal pulp in only 1.7% of participants, reversible pulpitis in 45.0%, irreversible pulpitis in 26.7%, and necrosis in 26.7%. According to the operative diagnosis, which was determined after pulp exposure, all the cases had some degree of pulp inflammation, and no normal pulp diagnosis was suggested. Reversible pulpitis, irreversible pulpitis, and pulp necrosis were suspected in 40.0%, 31.7%, and 28.3% of cases, respectively (Table 2).

**Figure 3:**
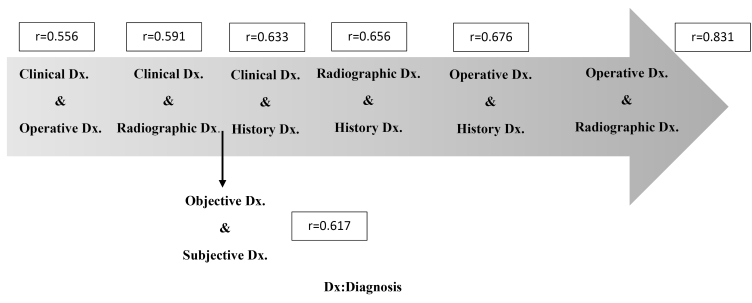
Correlation between clinical, radiographic, history-based diagnosis, and operative diagnosis

 Exploring the possible association between different sources of the pulpal diagnosis revealed the strongest correlation between the operative diagnosis and radiographic diagnosis (r = 0.831, *P* < 0.001) (Table 3, Figure 3). According to the rules for interpreting the size of Spearman’s correlation coefficient (Table 1), this correlation was rated very strong.

**Table 3:** Association of the diagnosis at each level of care

**Variable **	**History-based diagnosis**		**Radiographic diagnosis**		**Clinical diagnosis**		**Operative diagnosis**	
	***r***	***P***	***r***	***P***	***r***	***P***	***r***	***P***
History-based diagnosis	.	.	0.656	< 0.001	0.633	< 0.001	0.676	< 0.001
Radiographic diagnosis	0.656	< 0.001	.	.	0.591	< 0.001	0.831	< 0.001
Clinical diagnosis	0.633	< 0.001	0.591	< 0.001	.	.	0.556	< 0.001
Operative diagnosis	0.676	< 0.001	0.831	< 0.001	0.556	< 0.001	.	.

 According to our results, a strong correlation was found between a) objective data-based diagnosis and subjective data-based diagnosis (r = 0.617, *P* < 0.001), b) clinical diagnosis and history-based diagnosis (r = 0.633, *P <*0.001), c) radiographic diagnosis and history-based diagnosis (r = 0.656, *P* < 0.001), and d) operative diagnosis and history-based diagnosis (r = 0.676, *P* < 0.001) (Table 3, Figure 3).

 According to our results, a strong correlation was found between a) clinical diagnosis and history-based diagnosis (r = 0.633, *P* < 0.001), b) radiographic diagnosis and history-based diagnosis (r = 0.656, *P* < 0.001), and c) operative diagnosis and history-based diagnosis (r = 0.676, *P* < 0.001) (Table 3, Figure 3).

 Therefore, the strongest correlation was found between the operative diagnosis and the radiographic diagnosis (r = 0.831, *P* < 0.001), while the weakest correlation was observed between the operative diagnosis and the clinical diagnosis (r = 0.556, *P* < 0.001).

## Discussion

 Precise diagnosis of pulp status in primary teeth requires a thorough dental pain history and a comprehensive examination of both oral and extraoral tissues to consider all signs and symptoms. It is also crucial to evaluate radiographs for any abnormal radiographic changes. The final step in pulp diagnosis involves an operative diagnosis based on the color and amount of bleeding. Therefore, it is essential to integrate all diagnostic findings to confirm an accurate diagnosis of pulpal status.^14^ The correlation of these different diagnostic data is substantial. However, this consistency was not studied. Therefore, we evaluated the correlation between clinical signs and symptoms, radiographic findings, and operative diagnoses of pulpal status in primary molars of 4‒8-year-old children attending the Department of Pediatric Dentistry. Children aged 4‒8 were selected for our study because, in this age range, primary teeth often have fully developed, unresorbed, or minimally resorbed roots essential for proper endodontic treatment.

 In the present study, the most correlated diagnosis with the operative diagnosis was the radiographic diagnosis. The effect of pulp inflammation caused by dental caries on surrounding tissues (PDL, interradicular bone, and periapical bone) can be easily detected by the clinician, especially if this inflammation has passed the mild stage. Therefore, it cannot be treated under restorative procedures, and pulp therapy seems to be mandatory. Pathologic changes are most often apparent in the furcation areas of the primary teeth. Bitewing radiographs are often more effective in detecting pathologic changes in posterior primary teeth. Extension of pulpal pathosis into the periapical tissues is manifested as pathologic bone and root resorptions. Radiographic findings are objective and are very helpful for clinicians to draw accurate conclusions about the pulp status rather than relying solely on the child’s or parents’ reports.

 Consistent with our findings, Ashkenazi et al^15^ concluded that periapical radiography is highly effective in diagnosing pulp status and guiding endodontic treatment. In their retrospective case‒control study involving 102 primary maxillary incisors, they aimed to identify radiographic changes associated with pulp infection in primary incisor roots and the developing permanent dental follicles. The study revealed that deep caries, pathologic resorption, loss of lamina dura, and radiolucency in furcation and periapical areas were statistically associated with the presence of a sinus tract.^15^

 Our findings were not consistent with those of Morankar and Goyal,^16^ which was conducted on primary mandibular second molars indicated for single-visit pulpectomy. They found that radiographic changes were not common in primary teeth indicated for pulpectomy. This variation can be justified by the different selection criteria of the studies. Baseline radiolucency was present in only 11.7% of the selected cases, all of which were seen in the furcation areas. The lower rate of radiolucency at baseline was due to the inclusion criteria, which excluded cases with abscesses, excessive mobility, and soft tissue swelling. However, our study has shown a higher prevalence of radiolucency at baseline in the furcation areas where cases with dental abscesses and swelling were included.^16^

 All cases in the present study exhibited deep caries associated with pulpal infection, resulting in more pronounced changes in both soft and hard tissues compared to the initial lesions detectable through radiographic examination. Our findings were consistent with those of Coutinho and da Rocha Costa.^17^ They found that the radiographs could accurately diagnose the decayed surfaces, but their specificity for diagnosing sound surfaces was low.^17^ Taravati et al^18^ also found that periapical radiography may offer rather valuable information with high reliability about the presence and extent of external root resorption in primary molars.

 Moreover, Subka et al^19^ evaluated the in vivo validity of diagnostic radiographs for detecting proximal caries in primary teeth, with histological validation. They demonstrated that radiographic examination was significantly helpful in detecting D3 lesions (dentinal caries). The diagnosis must be confirmed by bitewing radiographs, as they are perfectly capable of detecting dentin lesions, unlike enamel caries.^19^ Interestingly, our findings also proved the diagnostic accuracy of radiographs because the majority of our cases had dentinal caries.

 Clinical diagnosis was the least correlated variable with operative diagnosis in the current study. Consistent with our results, Giuroiu et al^20^ reported that the correlation between clinical and histological diagnoses was relatively weak in both acute and chronic pulpitis. Mejàre et al^2^ also mentioned that there is insufficient data to determine an association between the presence and duration of clinical symptoms and the degree of pulp inflammation. The overall evidence was inadequate to assess the value of heat/cold pulp tests in determining the pulp status.^2^ This finding was not consistent with the other studies investigating the correlation between histological findings and clinical diagnosis. Naseri et al^21^ and Ricucci et al.^22^ found a good agreement between clinical and histological pulp diagnosis, particularly for cases with no irreversible pulpitis. Ricuuci et al^22^ assessed the reliability of the clinical diagnosis of irreversible pulpitis, reversible pulpitis, or healthy pulp, and the agreement between the findings of histological examination. They found that the clinical diagnoses correlated with the histological diagnosis of normal pulp and reversible pulpitis in 96.6% of samples, whereas the correlation for irreversible pulpitis was lower, at 84.4%.^22^ To elaborate on this controversy, it should be noted that the value of the examination relies on the patient’s ability to precisely indicate their present condition, which pediatric patients are not completely capable of.The mentioned studies were carried out on the permanent teeth of adults. The most probable reason for this difference can be the developmental and psychological differences between pediatric patients and adults. Children may be limited in their ability to provide accurate history and localize pain, and therefore, information attained from pulp tests in young children is mostly unreliable.

 The inability of children to describe the symptoms makes the precise diagnosis of pulp status one of the clinical challenges in pediatric dentistry. Language insufficiency or cognitive sophistication in children reduces the reliability of subjective pain reports.^14^ False-positive and false-negative responses are commonly seen with pulp sensitivity tests, especially when the examined teeth are immature. On the other hand, responses given by some children may be exaggerated due to their fear and anxiety. Evaluation of pulp vitality is complicated, particularly when dealing with pediatric patients, as their response may not be reliable. Furthermore, their anxiety may be exaggerated while performing the test.^5^ Therefore, the data gained from different sources in children could not always be consistent.

 Dental fear is one of the factors that affects the validity of the clinical data collected from children. Alshoraim et al^23^ demonstrated a significant relationship between dental fear, young age, and irregular and symptomatic dental visits, which was also the case in most of our patients.

 Our findings on the correlation between clinical and operative diagnoses align with those of Nagarathna et al’s^23^ study on the reliability of pulp sensitivity tests for primary teeth. Their study concluded that pulp testing in children under 10 is unreliable due to variability in children’s anxiety levels. Since these tests rely on subjective responses, they can yield false positives or false negatives if leading questions are asked. Additionally, the unpleasant stimuli produced by the tester may affect the child’s level of cooperation. However, these tests are found to be accurate for adults. Raoof et al^25^ evaluated the clinical, radiologic, and histologic correlations in the diagnosis of pulpitis, and similar to our results they found inconsistencies between clinical, radiographic, and histologic deductions, highlighting that effective clinical practice necessitates consideration of all these discrepancies.

 The correlation between clinical and radiographic diagnoses was moderate (r = 0.591, *P* < 0.001). Currently, the primary basis of clinical pulp diagnosis is mostly the pain duration reported by the patient (subjective) after thermal/electrical stimulation of the tooth. This can be considered a barrier to realistic speculation of the pulp status.^26^ The present available scientific data regarding the precision and reproducibility of diagnostic tests to assess pulp status is limited.^27^ However, the collection of radiographic findings does not require the patient’s report of the condition; instead, it relies solely on the clinician’s expertise to differentiate between pathologic and normal radiographic views. The most critical factor in radiographic interpretation is the clinician’s experience, as the detection of subtle radiographic changes is predominantly based on expert judgment.^28^ With the presence of an experienced clinician and the preparation of high-quality radiographs, it is possible to be sure that existing radiographic changes caused by dental pulp inflammation will be detected. However, vague reports of the severity and nature of pain by a young child that are the basis of routine pulpal sensitivity tests and clinical examinations can be confusing even to a skilled pediatric dentist.

 Our data demonstrated that the correlation between the history-based diagnosis and the operative diagnosis was stronger than that between clinical and radiographic diagnoses. Young children are often unable to provide an accurate account of their dental history. Therefore, in the present study, which included patients aged 4‒8 years, we primarily relied on parental reports to document symptoms. Parents were asked to report instances of spontaneous pain, nocturnal pain, referred pain, and sensitivity to cold, heat, and sweet snacks experienced by their children. This approach was logical, as parents could better recall and describe their child’s pain history without the pressure of the clinical environment. Clinical findings were based on the children’s subjective reports, whereas pain history was mainly derived from the parents’ accounts, who possess more developed communication skills.^29^ Another issue in documenting dental history is that sometimes patients, especially children, are naturally asymptomatic; consequently, pain may not be revealed in the patient’s anamnesis.^30^ Cisneros-Cabello and Segura-Egea^31^ assessed the correlation between the findings of histologic examination of pulp biopsy specimens and patients’ subjective symptoms and objective signs to determine the sensitivity, specificity, and reliability of signs and symptoms in adults. The results indicated that a history of previous pain, spontaneous pain, and prolonged pain in response to cold stimuli was significantly more common in patients with chronic pulpitis compared to those with transitional pulpitis. They also noted that “spontaneous pain” exhibited the strongest correlation with terminal pulpal status, demonstrating the highest reliability, acceptable sensitivity, and good specificity.^31^ To the best of our knowledge, no study has investigated the accuracy of the dental pain history provided by the parents in pediatric dentistry.

## Conclusion

 Based on the results, it can be concluded that clinical pulpal diagnosis had the least correlation with the operative pulpal diagnosis in primary molars. The highest correlation was observed between the operative pulpal diagnosis and the radiographic pulpal diagnosis. It seems that less subjective data exerts a higher correlation with the operative diagnosis.

## Limitations of the study

 This study mainly focused on the diagnostic data routinely collected in clinical practice. However, the gold standard for pulp status diagnosis is still a histopathologic examination of the pulp specimens. Therefore, it is suggested that future studies evaluate the correlation between histopathologic pulp diagnosis and other diagnoses mentioned in the present study.

 The recent consumption of analgesics or antibiotics in the study groups was not considered, which may have masked some clinical findings.

 The findings of this study can be further evaluated across diverse ethnic and cultural contexts to determine their generalizability. Responses to induced stimuli in pulp tests, as well as perceptions of dental treatments, may vary among children from different populations, which can influence the quality of subjective reporting. The authors recommend additional research to assess the reliability of diagnostic methods, considering factors such as tooth type (primary or permanent), patient age, and the histological stage of the disease.

## Competing Interests

 The authors declare that they have no competing interests and no conflicts of interest.

## Ethical Approval

 Ethical approval for this study was obtained from the Research Ethics Committee of Tabriz University of Medical Sciences (IR.TBZMED.REC.1399.445). Informed consent was obtained from all subjects and from parents and/or legal guardians of children in the study.
